# Book Reviews

**Published:** 1916-01

**Authors:** 


### BOOK REVIEWS

Books mentioned may be inspected at and ordered through this office. So far as possible, books received in any month will be reviewed in the issue of the second month following.

**Zeitkinder,** Dr. Henry Jones Mulford, of Buffalo, author and publisher, 107 pages, $1.00.

While this play is described as one to be read, the difficulties of the role of the infant and small child do not appear insuperable for the stage or the film-drama. As the work is pre-eminently one with a purpose, we hope that this purpose will be given wide publicity by such actual dramatization. The story is of a modern city child, with a sensible father yielding, as most of us do, to feminine authority in domestic matters and a pretty, somewhat foolish mother. This combination would not be so bad were it not for the maternal grandmother, superstitious, over-confident and sceptic of medical skill, who is abetted by adult friends of the superficial society type, inclining to osteopathy and somewhat hostile to medical authority. One of our patients once said: "Doctor, my wife is a ——— fool. Any woman who is any good is a ——— fool." While not agreeing with this dictum in its extensive meaning, it contains a germ of practical wisdom. The medical plot of the story is a series of minor neglects and lack of wisdom, culminating with death from septic meningitis after otitis media. The real hero of the story is Dr. North who does not make "the fatal mistake of being polite to his patients" but who is a sincere, skillful practitioner. We are disposed to take issue with him as to the unhygienic conditions of the better residence district of a city, as to the wisdom of sending a small child who has the tendencies noted to the country, and as to the policy of keeping normal children out of school till the age of 8 or 10. Of course, in particular conditions of a real case, all of these objections might be contrary to the interests of the patient but we have reference to the general inferences which the public will naturally draw.

The date of birth of the child, Hallowe'en, affords the opportunity for developing the superstitious side of the mother and grandmother and for a prologue in which Dr. North, walking home after a break down of his car, encounters Lucifer, and imps of Ignorance, Selfishness, Doubt and Crime, conspiring against the child. This personification seems to us a particularly strong point of the play, as tending to convince superstitious persons of the truth back of their notions, though Dr. North subsequently alludes to the scene as a dream.

**37th and 38th Annual Reports of the Charity Organization Society of Buffalo, 1915.**

This large pamphlet gives in detail, a description of the various activities of the C. O. S. and a financial statement which indicates the ultimate economy of this form of charity and the urgent need of support.

---

**Annual Report of the Department of Health of Los Angeles.** Year ending June 30, 1915.

While the activities of the Health Dept. do not differ materially from those of other well organized cities, it should be recognized that, in this instance, there has been the difficult factor of adjustment to a very rapid growth of population. From 1890 to 1902, there was a very gradual increase—for some years indeed, a decrease—from 80,000 to 100,000. From this time, the city has increased fivefold, to about 500,000. Tuberculosis has been a marked evil. 17.43% of all deaths occur from this disease or 2:1000 of population. This is about 1¾ times the tuberculosis problem with which the average eastern city has to deal. A favorable item is that the deaths from this disease have not increased as fast as the population although the cases have increased more rapidly. That tuberculosis is mainly an introduced factor is apparent from the fact that, among 1000 deaths, in the year, only 111 were of natives of the city and only 27 of natives of the state. We note also that 24 cases of small-pox occurred, with no mortality. Of these, 14 had never been vaccinated and 3 more never till after exposure, while 7 had not been vaccinated since infancy. The general death rate was only 11.47:1000 population, though the qualification that this ratio is based on an estimated population of 500,000, leads us to think that the Health Dept. shares to some degree the general scepticism as to the actual degree of growth of the city.

---

**Medical Directory of N. Y., N. J., and Ct., 1915,** published by the Medical Society of the State of N. Y.

This directory, now in its 17th volume, is one of the most valuable activities of the State Society. The present volume is bound in stiff paper and is slightly smaller than former editions. The number of physicians in the state has increased somewhat but has declined relatively to the population. In 1910, 13,474 physicians were listed, a ratio of 1:676.3 of the 9,113,000 population, This year the physicians number 14,-156, a ratio of 1:692.3 of the 9,700,000 population.

**Physicians' Visiting List** (Lindsay & Blakiston) for 1916, 65th year. P. Blakiston's Son & Co., Philadelphia: for 25 patients per day or week, $1.25; for 100 patients $2.50; also prepared for 50 and 75 patients.

**Medical Record Visiting List for 1916,** Wm. Wood & Co., N. Y. for 60 patients.

Both of these visiting lists are neatly bound, with pocket and pencil and contain a large amount of information useful in emergencies. We are strictly neutral in regard to them, both being admirably adapted to their purpose.

---

**Transactions of the American Otological Society,** 48th annual meeting, Niagara Falls, Ont., June 3 and 4, 1915. Vol. 13, Part 3. Published by the Society, Mercury Pub. Co., Printers, New Bedford, Mass.

Copies may be secured from the Secretary Dr. John B. Rae, 247 W. 70, N. Y. This contains lists of members and officers, minutes of the meeting and a collection of valuable papers, some illustrated.

---

**Progressive Medicine,** a quarterly digest of advances, discoveries and improvements in the medical and surgical sciences. Edited by H. A. Hare and L. F. Appleman, Philadelphia. Published by Lea & Febiger, Philadelphia, $6 per annum.

This volume deals with the Digestive Tract, Kidneys, G. U. diseases, various Surgical problems and ends with a Practical Therapeutic Referendum.

---

**The Medical Clinics of Chicago.** Volume I, Number III, (November 1915). Octavo of 200 pages, 23 illustrations. Philadelphia and London: W. B. Saunders Company, 1915. Price per year. Paper, $8.00. Cloth, $12.00.

---

**Anatomy of the Brain and Spinal Cord,** with Special Reference to Mechanism and Function. Harris E. Santee, M. D., Ph. D., Chicago. P. Blakiston's Son & Co., Philadelphia. 5th edition, 474 pages, 158 illustrations, 46 in colors, $4.00.

In each region, the description passes from gross anatomy to neurons, the relation of the anatomy to physiologic function being always kept in mind so that the arbitrary nature of anatomic study is minimized. Embryologic development is included wherever it will assist in the comprehension of the adult forms, the special embryologic chapter included in previous editions, having thus been distributed throughout the work. The work has also been brought up to the most recent discoveries. The Basle Nomenclature of Anatomy has been followed almost without exception.

---

**The Nose, Throat and Ear,** their Functions and Diseases. Ben Clark Gile, M. D., Philadelphia. Published by P. Blakiston's Son & Co., Philadelphia. 456 pages, 131 illustrations, 8 in colors, $2.75.

The epigrammatic sub-title "A Treatise upon the Breath-Road, Food-Road and Accessory Organs" is worth noting. While the general arrangement of the work does not differ much from accepted standards, the same epigrammatic emphasis of facts is found throughout. The final chapter on Deaf-Mutism though brief, is exceptionally interesting.

---

**The Practical Medicine Series,** 10 volumes on the year's progress in medicine and surgery, under the general editorial charge of Charles L. Mix, A. M., M. D., Chicago; The Year Book Publishers, $10.00 for the series.

Vol. 6, General Medicine, edited by Frank Billings, M. S., M. D., and J. H. Salisbury, A. M., M. D., Chicago, 359 pages, $1.50 separately.

Vol. 7, Obstetrics, edited by Joseph B. DeLee, A. M., M. D., and Herbert M. Stowe, M. D., Chicago, 230 pages, $1.35 separately.

The books of this series are well illustrated and are indexed by topics and authors, with references to periodic literature in foot notes. On the whole, it is the most complete and most satisfactory review of medical literature published.

---

**Municipal Ordinances, Rules and Regulations Pertaining to Public Health.** Reprint No. 273 from Public Health Reports.

This is a compilation made under the supervision of the Surgeon General of the U. S. P. H. S. and contains ordinances, etc., adopted during 1914 by cities of the U. S. having a population of 10,000 or more in 1910. Aside from its immediate and direct value, the size of this book (650 pages), the repetitions, omissions and inconsistencies as we compare the ordinances for different places, point clearly to the desirability of unified and centralized authority for this country.

